# Evaluation and Formulation of Hybridized Biobased Precursors as Anticorrosive Surface Coatings

**DOI:** 10.1021/acs.biomac.5c02310

**Published:** 2026-03-24

**Authors:** Emre Kinaci, Sarah A. Salazar, Giuseppe R. Palmese, Joseph F. Stanzione

**Affiliations:** † Department of Chemical Engineering, 3536 Rowan University, 201 Mullica Hill Rd, Glassboro, New Jersey 08028, United States; ‡ Advanced Materials & Manufacturing Institute (AMMI), 3536 Rowan University, 107 Gilbreth Pkwy, Mullica Hill Rd, Glassboro, New Jersey 08062, United States

## Abstract

This study focuses on the synthesis of an oligomeric diepoxy resin (EVAC) derived from two diverse biomass sources, cashew nutshell liquid and lignin, and its evaluation as a major component in an anticorrosion surface coating. EVAC structural verifications were performed via spectroscopic and chromatographic methods. EVAC was mixed with diglycidyl ether of bisphenol A (EPON-828) at varying ratios and cured with 5,5′-methylene difurfurylamine (DFDA) and diethylmethylbenzenediamine (Epikure W). Thermally cured blends containing DFDA demonstrated improved moduli and char yields relative to those containing Epikure W. Although the replacement of EVAC with EPON-828 resulted in a reduction in the glass transition temperature and the strength, desired hydrophobicity, flexibility, and adhesion for coatings applications were imparted. Additionally, biobased DFDA demonstrated improved coatings and corrosion performance relative to Epikure W without significantly affecting network performance. Markedly, EVAC/DFDA-based formulations demonstrated high gloss, substrate compatibility, adhesion (5B), impact resistance (>160 lb.-ft), and durability characteristics.

## Graphical abstract

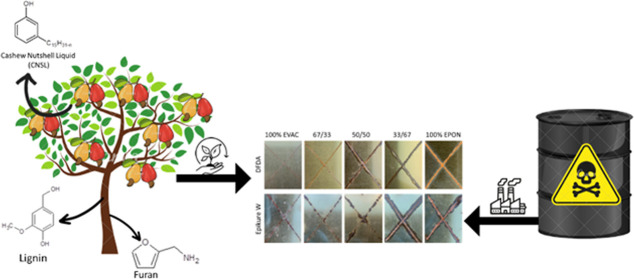

## 1 Introduction

Epoxy resins are an important class of thermoset polymers that are characterized by the presence of more than one 1,2 epoxide group (oxirane ring).^1^ They are commonly used in protective coatings, adhesives, reinforcements, and structural composites due to their ability to impart desired strengths, dimensional integrity, barrier properties, surface adhesion, moisture, and chemical resistance when properly cured.^2^ In addition, epoxies are generally characterized by their susceptibility to both electrophilic and nucleophilic attack, making the resin reactive to a wide range of chemical reagents. Epoxy resins can be catalytically homopolymerized or reacted with curing agents (i.e., curatives and hardeners), both of which allow epoxide ring opening and promote subsequent chain extension, accompanied by an overall molecular weight increase.^3^ The properties of cured networks vary with the specific combination of resin type, curing agent, and associated curing mechanism. The selection of curing agent determines not only the ultimate application of an epoxy system but also its curing temperature, cured structure, reactivity, and end-use properties. Common curing agents are aliphatic and aromatic hydrocarbon derivatives such as amines, phenols, alcohols, carboxylic acids, acid anhydrides, and thiols.^4^ Among them epoxy-amine systems are further characterized by their fast and low temperature cure, low curing shrinkage, high gloss, toughness, and surface adhesion, along with good processability and compatibility with a wide range of organic and inorganic fillers, making them a leading candidate for CASE (coatings, adhesives, sealants, and elastomers) applications.^5^

Epoxy resins are typically synthesized via the epoxidation of Petri-based bisphenol precursors, such as bisphenol A (BPA), leading to economic and environmental concerns. Petroleum is a finite resource, and BPA derivatives are also well-known endocrine disruptors that can cause serious health disorders, largely in younger children.^6,7^ Exposure to BPA has been shown to have adverse effects on the brain, immune system, reproductive system, and metabolic processes thereby their use in infant products is strictly restricted by environmental agencies.^8^ The growing economic and environmental concerns have led to the development of sustainable polymers made from readily available renewable resources. In an attempt to potentially replace BPA, many studies have been conducted where epoxy monomers and curing agents have been synthesized from biobased precursors such as ligno-cellulose, distilled cashew nutshell liquid (cashew nut shell liquid (CNSL)), vegetable oils, cannabis derivatives, and terpenoids.^9–12^

Cardanol is a phenolic lipid that is produced via vacuum distillation of CNSL found inside the shell of the cashew nut.^13^ The cardanol molecule provides a combination of a rigid and reactive phenolic group, and a flexible and hydrophobic C_15_ alkyl side chain connected on meta-position with varying unsaturation points.^14,15^ These characteristics make cardanol an excellent candidate for monomer synthesis, with improved functionality to produce high-performance coating materials. Studies report the application of cardanol in a wide range of thermoset coating applications such as benzoxazines, polyurethanes, vinyl esters, epoxy resins, and phenalkamine curing agents.^16–19^

Lignin is one of the most abundant biopolymers in nature, found in the cell walls of plants to give strength, rigidity, and reinforcement to the structures of plants.^20^ It is an industrial waste byproduct obtained from the paper and pulp industry—produced at a rate of over 50 million tons annually.^21^ Vanillin from lignin is currently being produced at industrial scales and accounts for 15% of its annual production. Because of this, and the environmental advantages associated with it, the use of vanillin has gained considerable interest, especially for epoxy resin synthesis.^22,23^ Additionally, vanillin derivatives, such as vanillic acid and vanillyl alcohol (VA), have been proven to be promising building blocks. VA has become attractive for epoxy synthesis because of the presence of an extra hydroxyl group that can be functionalized for thermoset resin synthesis. It has been reported that VA can be used as a platform chemical to synthesize difunctional epoxy monomers and the resulting cured epoxy resins demonstrated promising characteristics and performance properties similar to certain BPA-based cured epoxy resins; however, such cured epoxy resins still lacked ease of processing and matching mechanical performances, mostly due to the lack of bisphenol character which is known to assist with both qualities.^24,25^

Biobased epoxy-amine coatings have been increasingly utilized in various corrosion protection applications, particularly as surface coatings. In a recent study, an isosorbide-based diepoxide was formulated with superhydrophobic SiO_2_ nanoparticles, hexadecyltrimethoxysilane (HTMS), and cured with a commercial silane-based amine (Dynasylan). The formulation was spin-coated onto glass and aluminum substrates. While the unmodified epoxy-amine formulation exhibited lower contact angle, surface roughness, and reduced corrosion inhibition efficiency, coatings modified with SiO_2_ and HTMS demonstrated enhanced corrosion resistance.^26^ In a similar study, cardanol-based oligomeric resins (NC-514 and NC-547) were thermally cured in the presence of 10% polydimethoxysilane (PDMS) on iron substrates using furfurylamine and diamino-*p*-menthane (DAPM) as curing agents. The addition of monofunctional furfurylamine significantly reduced both coating performance and corrosion resistance due to a lower cross-link density. However, the inclusion of PDMS improved adhesion, corrosion potential, and corrosion current density.^27^ Another study involved epoxidizing technical cashew nutshell liquid (CNSL) through its side chain and applying it as a one-component system on steel panels, using an imidazolium catalyst and over 30 wt % of solvents (acetone and xylene), followed by curing at 150 °C. These coatings exhibited good adhesive properties and high impedance modulus, with minimal delamination during testing. In a similar effort, epoxidized soybean oil (ESO) and tannic acid (TA) were mixed in ethanol at varying molar ratios (ESO/TA from 1:0.5 to 1:2.5) and applied on carbon steel by using both film applicator and spray-coating methods. The coatings were cured at 170 °C for 18 h and demonstrated excellent adhesion (5B rating). While all formulations offered satisfactory corrosion protection, those with medium to low TA content exhibited the best corrosion resistance.^28^ Notably, the use of excessive solvents and high curing temperatures remains a significant drawback of these systems.^29^ Several studies have also explored the incorporation of inorganic fillers such as graphene and titanium oxides into bioderived epoxy-amine systems to enhance corrosion resistance. For example, a cardanol-based oligomeric diepoxide (NC-514) was formulated with 3-glycidoxypropyltrimethoxysilane (GPTMS)-functionalized Ag–TiO_2_ (GAgT) nanoparticles in varying ratios and applied to mild steel substrates. The presence of GAgT within the cardanol epoxy (CE) matrix enhanced the hydrophobicity and lowered the surface free energy, reducing interfacial interactions with microbes. An optimal GAgT loading of 3 wt % in CE provided high corrosion resistance, maintaining impedance values up to 1097 Ω after 21 days in microbial coculture medium.^30^ In another study, modified gelatin (MG) and graphene oxide (GO) were used as additives in a commercial epoxy-amine coating system. Gelatin and polyamine (DETA) were premixed in water to produce MG, while GO was dispersed ultrasonically in water and then incorporated into the MG matrix to form MG-GO. Coatings prepared using this MG-GO composite showed a 59% improvement in corrosion resistance compared to coatings containing MG alone.^31^ Similarly, epoxy EPON-828 polyamine epoxy-amine networks were incorporated with modified nanoclay particles at varying ratios (1, 3, and 5 wt %) and evaluated for corrosion resistance via salt spray and electrochemical impedance spectroscopy. Although all the formulations showed high adhesion to metallic substrate (5B), >3% nano clay loaded samples were found to have significantly lower water uptake and reduction in degradation and blistering density.^32^

As it is pointed out, epoxy-based surface coatings are intrinsically hydrophilic and rely on excessive formulations with volatile solvents, adhesion promoters, fillers like nanoparticles, graphene, or the use of corrosion inhibitors in significant amounts. In this study, we evaluated neat biobased epoxy-amine formulations as anticorrosion coatings, formulated without inorganic fillers, adhesion promoters, or volatile solvents. The bulk synthesis of a potential biobased BPA replacement was achieved via the oligomerization of cardanol (C) and VA, using a recyclable heterogeneous strong acid catalyst and without the use of toxic phenol or formaldehyde. The resulting prepolymer [vanillyl alcohol cardanol (VAC)] was subsequently epoxidized using epichlorohydrin to produce an epoxidized resin (EVAC) with the potential to meet regulatory standards as a safer, biobased alternative. Structural characterization of VAC and EVAC was conducted using ^1^H and ^13^C nuclear magnetic resonance (NMR) spectroscopy, while the extent of oligomerization was confirmed through advanced polymer chromatography (APC). EVAC was blended with petroleum-derived EPON-828 (epoxy equivalent weight: 186.1 g/eq) at varying weight ratios (0%, 33%, 50%, 66%, and 100%), and cured with stoichiometric amounts of either a biobased furan diamine (DFDA) or a conventional aromatic curing agent (Epikure W). The resulting epoxy–amine systems were evaluated as potential surface coating formulations and benchmarked against their petroleum-derived phenolic analogues. Thermal and mechanical properties of the cured networks were assessed via thermogravimetric analysis (TGA), dynamic mechanical analysis (DMA), tensile testing, and contact angle measurements. While full replacement of EPON-828 with EVAC resulted in decreased glass transition temperature (*T*_g_) and Young’s modulus (e.g., from 3.2 to 0.3 GPa and 102 to 19 °C for DFDA; 2.8 to 0.9 GPa and 172 to 40 °C for Epikure W), enhancements in flexibility and hydrophobicity were observed, attributed to the polar and flexible aliphatic side chains of EVAC. Additionally, formulations based on the furan-derived DFDA exhibited higher modulus, char yields, increased cross-linking densities, and lower *T*_g_ values compared to Epikure W systems at equivalent EVAC loadings. Thin-film coatings (5 mil) were applied to mild steel panels using a bird applicator and evaluated for coating performance, including impact resistance, crosshatch adhesion, Shore hardness, flexibility, gloss, and environmental resistance. Notably, EVAC/DFDA-based formulations demonstrated high gloss, excellent substrate compatibility, outstanding adhesion (5B), high impact resistance (>160 lb-ft), and durability under environmental stress, achieved without the use of expensive and toxic adhesion promoters, corrosion inhibitors, pigments, or additives. The superior performance is attributed to EVAC’s unique molecular structure, which imparts chemical resistance, polarity, and flexibility, while the enhanced adhesion is proposed to result from the furan rings in DFDA. Cure schedules were systematically varied to examine their influence on final coating properties. This resin could meet regulatory requirements under frameworks such as the US Toxic Substances Control Act and the EU Registration, Evaluation, Authorization and Restriction of Chemicals (EU-REACH), which impose significant costs and restrictions on monomeric epoxy resins classified as toxic substances. In contrast, oligomeric resins are considered polymers and are exempt from such regulations, offering cost-effective alternatives for industrial applications.^33–35^ Given the increasing demand for environmentally responsible, low-viscosity epoxy oligomers with high performance under mechanical and environmental stress with minimal formulation requirements, materials such as the developed EVAC resin are of significant interest. This work also advances a mechanistic understanding of biobased functional coatings by correlating chemical structure with coating and protection behavior.

## 2 Experimental Section

### 2.1 Materials

VA (4-hydroxy-3-methoxybenzyl alcohol, Cas#:498-00-00) was purchased from Sigma-Aldrich (≥98%). High purity low odor cardanol (NX-2026, Cas#:37330-39-5) was supplied by Cardolite Corporation (Bristol, PA) and used as received. Epichlorohydrin (>99%) and TEAB (tetraethylammonium bromide, >99%) were purchased from Acros Organics. Amberlyst 15 catalyst, aqueous sodium hydroxide solution (50%), and toluene were purchased from Fisher Scientific. Tetrahydrofuran (THF, >99.9%), deuterated chloroform (CDCl_3_, >99.9%), sodium chloride (NaCl, 100%), and magnesium sulfate (MgSO_4_, 100%) were purchased from MilliporeSigma (Milwaukie, MI). Argon gas used in the synthesis reactions was obtained from Airgas (99.99%). EPON-828 (diglycidyl ether of BPA—DGEBA, epoxide equivalent weight (EEW) = 186.1 e/eq), and Epikure W (diethyl toluene diamine, AHEW = 44.5 g/eq) curing agent were obtained from Hexion. DFDA (5,5′-methylene difurfurylamine, AHEW = 51.5 g/eq) curing agent was prepared as it is previously reported.^36^ The representative chemical structures of the functional materials used for the coating formulations investigated herein are presented in Scheme 1.

**Scheme 1:**
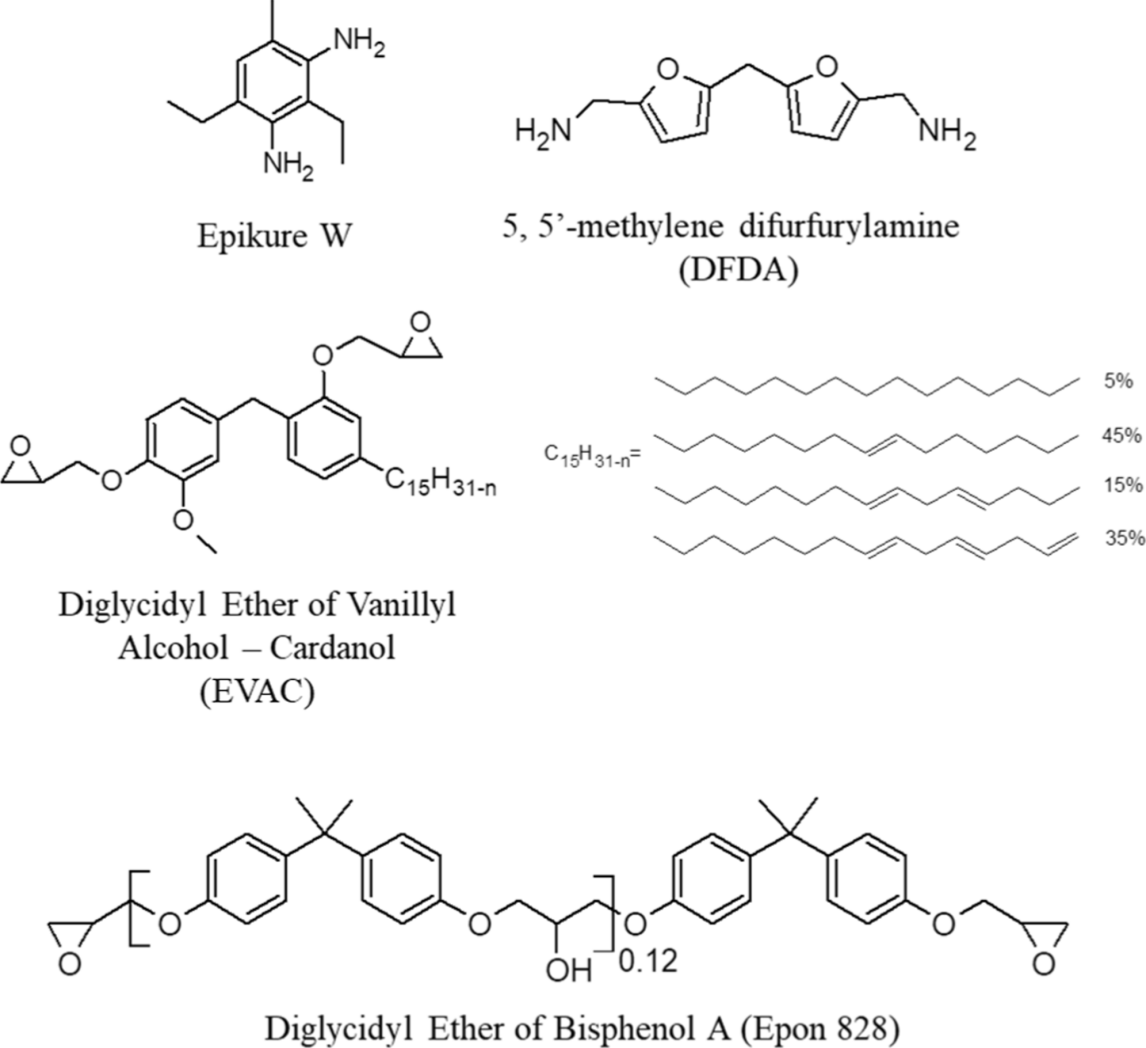
Structures of epoxy resins and amine curing agents used in this study

### 2.2 Methods

#### 2.2.1 Preparation of Vanillyl Alcohol Cardanol Resin

A 2 L four-neck round-bottomed flask equipped with a mechanical stirrer, gas inlet, temperature probe, and Dean–Stark assembly was charged with 330 g of cardanol (1.1 mol) and 48.4 g of Amberlyst-15 catalyst (10 wt %). Contents were mixed until the temperature reached 90 °C with a continuous argon purge. At 90 °C, 154 g of VA (1 mol) was divided into 4 equal portions and charged over a 4 h period. After complete addition, the reaction continued at 90 °C for at least 26 h until all the VA was consumed via the electrophilic aromatic condensation reaction with excess cardanol and until no more water formed in the Dean–Stark assembly. After the reaction was complete, the catalyst was filtered and washed with acetone to be recycled. 420 g of a transparent-orange liquid resin, dubbed as VAC was obtained (86% yield, viscosity at 25 °C = ∼1000 cP).

#### 2.2.2 Preparation of the Epoxidized VAC

A 2 L four-neck round-bottomed flask equipped with a mechanical stirrer, condenser, temperature probe, addition funnel, and gas inlet was charged with 480 g of VAC liquid resin (∼1 mol), 462 g of epichlorohydrin (5 mol), and 2.4 g of TEAB catalyst (0.5 wt %). The mixture was mechanically stirred for 2 h at 80 °C with a constant argon flow. The reaction was cooled to 75 °C, and 168 g of 50 wt % NaOH_(aq)_ solution (2.1 mol) was added to the flask dropwise over a 2 h period. After complete addition, the reaction was held at 80 °C for 1 h and then 95 °C for another 1 h. After the reaction was complete, the product was dissolved in 350 g of toluene and washed with brine (15%) twice and with DI water once. Toluene and excess epichlorohydrin were removed through vacuum distillation after MgSO_4_ drying. 500 g of a transparent orange liquid resin was obtained (83% yield, viscosity at 25 °C = 314 cPs). The combined two-step synthetic process is illustrated in Scheme 2.

**Scheme 2:**
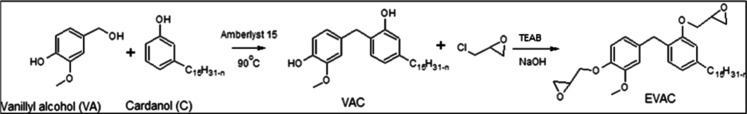
Illustration of the Combined Synthetic Process to Make EVAC

#### 2.2.3 Monomer Characterization

The molecular weight distribution of VAC and EVAC liquid resins was collected on a Waters Acquity APC instrument with a refractive index (RI) detector. Samples were prepared by dissolving the liquid resin in Optima grade THF (10 mg/mL). A series of 4.6 × 150 mm ACQUITY APC columns (XT 450 2.5 μm, XT 125 2.5 μm, and XT 45 1.7 μm) were used at 40 °C, and samples were run in THF at a flow rate of 0.6 mL/min.

The modified resins were characterized via proton and carbon NMR (^1^H/^13^C NMR) spectroscopy using a Varian 400 MHz NMR spectrometer. Samples were prepared by dissolving the liquid resins in deuterated chloroform (CDCl_3_) at a concentration of 10–15 mg/mL, and 32 scans were collected per sample at 298 K at 90° pulse width.

The epoxy content of EVAC resin is expressed as EEW and can be defined as the weight of resin containing one equivalent gram of epoxide (g/eq). A standard method for analysis of epoxy content is a manual titration method that follows ASTM D-1652.^37^

#### 2.2.4 Resin Formulation and Extent of Cure

Epoxy blends of EVAC and EPON-828 at different weight ratios (0, 33, 50, 67, and 100% EVAC) were mixed and degassed in a Thinky ARE planetary mixer for 15 min after the addition of a stoichiometric amount of DFDA or Epikure W. The homogenized epoxy-amine mixtures were cast into rectangular rubber molds with approximate dimensions of 35.0 × 13.0 × 3.0 mm^3^ for DMA testing and dog-bone shaped rubber molds (ASTM, type IV) for tensile testing. The blends were kept at laboratory temperature for 24 h. After 24 h, the epoxy systems with DFDA were thermally cured for 6 h at 120 °C, 12 h at 160 °C, and then post-cured for 3 h at 180 °C. The epoxy systems with Epikure W required longer cure times at higher temperatures. These blends were thermally cured for 12 h at 90 °C and 12 h at 180 °C to ensure complete epoxy-amine conversion. The fully cured samples were cooled to laboratory temperature, demolded, and sanded to obtain uniform dimensions for DMA and tensile testing.

The extent of cure for all epoxy-amine blends was measured using a Nicolet iS50 FT-IR spectrometer in the near-infrared (NIR) region (4000–8000 cm^–1^). Before and after cure samples were examined at 8 cm^–1^ resolution while collecting 32 scans per spectrum at ambient conditions. The peak at 4530 cm^–1^, which corresponds to vibrations related to oxirane groups, was tracked along with peaks corresponding to vibrations related to amines, primary amines at 5900 cm^–1^, and primary and secondary amines at 6600 cm^–1^. Equation 1 was used to calculate the extent of cure. In eq 1, ABS­(*t*) represents the reduced absorbance of the relevant IR peak before and after cure.

1 $$\alpha =1-\frac{ABS\left(t\right)n}{ABS\left(t=0\right)n}$$

#### 2.2.5 Viscosity and Gel Time

Rheological behavior of the epoxy-amine resins was determined via a TA Instruments discovery hybrid rheometer. Shear viscosity (η) at 25 °C was measured as a function of the shear rate. Liquid resin was placed directly on a 40 mm parallel plate geometry, and the Peltier plate was kept at 25 °C. The shear rate was increased logarithmically from 1 s^–1^ to 100 s^–1^ with 5 points per decade and decreased from 100 s^–1^ to 1 s^–1^ with 3 points per decade to observe if the liquid resins exhibited non-Newtonian behavior. The gel time (*t*_gel_) at 25 °C of each epoxy system was also determined using the same instrument by tracking the storage modulus (*G*′) and loss modulus (*G*″) of each blend until crossover. For this measurement, a 20 mm parallel plate was used with an oscillation frequency of 1 rad s^–1^ and a strain of 0.1%. Only epoxy systems containing DFDA were tested for the laboratory temperature gel time measurements, with three replicates for each formulation.

#### 2.2.6 Polymer Characterization

##### 2.2.6.1 Thermogravimetric Analysis

TGA of the cured resins was measured using a TA Instruments Discovery TGA 550. Sample sizes of 8–10 mg were placed in an aluminum pan and heated from −50 to 800 °C at a heating rate of 10 °C min^–1^ in an inert (N_2_) environment. The initial decomposition temperatures at 10% weight loss (*T*_10%_), the temperature where 50% decomposition occurs (*T*_50%_), as well as char residue at 700 °C, were collected from TGA thermograms. Each specimen was tested in triplicate.

##### 2.2.6.2 Dynamic Mechanical Analysis

DMA tests were performed on a TA Instruments Discovery DMA 850. Samples had a rectangular shape (35 mm × 11.5 mm × 2.5 mm) and were characterized using a single cantilever geometry. Test parameters included an amplitude of oscillation of 7.5 μm (displacement), a frequency of 1 Hz, and a Poisson’s ratio of 0.35. The post-cured formulations were thermally scanned from −50 °C by 2 °C min^–1^ rate to well above the glass transition temperature of the tested sample to obtain storage modulus (*E*′), loss modulus (*E*″), and tangent delta (tan δ) values as a function of temperature. The temperatures at the peak of the loss modulus and tan δ thermograms were reported as the glass transition temperature (*T*_g_) of the cured resin. The cross-link density (*v*) and molecular weight between cross-links (*M*_c_) of the cured resins were calculated using the theory of rubber elasticity above 60 °C of the *T*_g_ (loss modulus) and the density of each sample using eq 2.^38^ In eq 2, *E*′ represents the storage modulus as obtained via DMA runs, *T* is the absolute temperature, and the *R* is the universal gas constant. The density measurements were performed via Archimedes’ principle according to the ASTM D792 standard test method.^39^ Each cured resin was run in triplicate to ensure consistency.

2 v=E′3RT

##### 2.2.6.3 Differential Scanning Calorimetry

Differential scanning calorimetry (DSC) thermograms of the cured resins were obtained using a TA Instruments Discovery DSC 2500. Sample sizes of 5–10 mg were prepared in hermetic aluminum DSC pans and subjected to a heating rate of 10 °C min^–1^ from −20 to 180 °C under a N_2_ purge. *T*_g_ of the post-cured formulations was obtained from the thermograms as a sudden slope change.

##### 2.2.6.4 Tensile Tests

Tensile properties of the cured resins were examined through tensile testing. An Instron 5966 mechanical testing system was used for the measurements with a 1 kN load cell. Type IV samples of each cured resin were tested following ASTM D-638 and tested at ambient conditions using 1 mm min^–1^ strain rate.^40^ An extensiometer with 5 in. gauge length was used during the test to accurately calculate the Young’s Modulus (*E*). At least six samples of each cured resin formulation were tested for consistency.

##### 2.2.6.5 Contact Angle Measurements

Surface hydrophobicity of the coatings was determined by using sessile drop contact angle experiments. Water was dropped on the cured resin surface at 25 °C. An LED light source was directed toward the sample to provide a sharp image of the drop on the sample surface. A FireWire camera and a 420 fps high speed Ethernet camera were used with a resolution of 656 × 150 pixels.

##### 2.2.6.6 Scanning Electron Microscopy

The surface morphology analysis of the cured resins was performed on a Phenom XL scanning electron microscopy (SEM) at ambient temperature. The fractured surfaces of the tensile bars were used for data collection. Samples imaged in Phenom XL were sputtered with gold using a Cressington 108 sputter coater. Images were taken at 10 kV using backscatter and secondary electron detectors.

#### 2.2.7 Preparation of Coatings

Coatings were prepared using a bird film applicator (1 mil) to cast the liquid resin blends onto mild stainless steel panels (Q Panels, QD 2 × 3.5 × 0.025 in^3^). All coatings were left at ambient conditions for 24 h and then separated to follow three separate curing schedules to observe the effect of forced curing on coatings’ performance and properties. After 24 h, one set was cured at ambient temperature for another 6 days (in total, a week), the second set was cured in a thermal oven for 4 h at 80 °C, and the third set was force cured (FC) to follow the same curing schedule for samples tested for thermomechanical and mechanical evaluations. The FC epoxy blends cured with DFDA were cured at 120 °C −6 h, 160 °C −12 h, and 180 °C −3 h, while epoxy blends cured with Epikure W were cured at 90 °C −12 h and 180 °C −12 h to ensure full epoxy-amine conversion.

#### 2.2.8 Coating Tests

##### 2.2.8.1 Gloss

The percentage gloss on the steel panels was measured via KSJ MG6 Stone Gloss Meter at angles of 20°, 60°, and 85° according to ASTM D-523.^41^

##### 2.2.8.2 Shore D Hardness

To determine Shore hardness, 1 in. thick samples were cured according to ASTM D-2240.^48^ A Shore D durometer was used, with measurements obtained in triplicate.

##### 2.2.8.3 Cross-Hatch Adhesion

Cured coatings were evaluated for their adhesive strength to the stainless steel substrates using cross-hatch adhesion with commercial transparent tape (Scotch Transparent Tape, 25 mm) according to ASTM D-3359.^42^ Cross-hatch cuts of 10 mm × 10 mm consisting of 100 squares of 1 mm × 1 mm dimensions were made on the coated steel panels. The adhesive tape was placed on the cut surface, and sufficient pressure was applied to remove all the air bubbles. The tape was pulled quickly in a direction perpendicular to the film. Observations were recorded for the removal of any coating squares. If no squares remained on the coating, then 0% adhesion of the coatings to the substrate was considered (0B). If no squares were pulled off by the tape, then 100% adhesion was considered (5B). Partial removal of the square represents adhesion values between 0B and 5B and gave a value of 1B, 2B, 3B, or 4B, depending on the count, all in accordance with ASTM D-3359.

##### 2.2.8.4 Impact Resistance

Impact resistance of the coatings was measured using a BYK-Gardner Impact Tester according to ASTM D-2794 (max height 127 cm, max load 4 lb.).^43^ The coated panels were placed at the bottom of the test unit, and the minimum height at which the dropping ball caused any crack in the dent was utilized to determine the impact resistance in ft-lb./in.

##### 2.2.8.5 Conical Mandrel Flexibility

Flexibility of the coatings was measured using a BYK-Gardner conical mandrel bender according to ASTM D-522.^44^ The coated dried panels were clamped in the mandrel and bent by the roller frame. The tested panels were visually examined for any cracks in the coating, delamination, loss of adhesion, or any other physical changes.

##### 2.2.8.6 Environmental Resistance

The environmental resistance properties of the coatings were analyzed by scribing an X in the middle section of the coatings that cured at ambient temperature (1–2 weeks) and exposing them to harsh environmental conditions. All the coated panels were kept outside under direct sunlight, rain, and high humidity in New Jersey, USA, during the months of July and August. Coatings were visually evaluated after 2 weeks. Any visual changes related to the blister formation, creeping, and swelling of the coatings were recorded.

## 3 Results and Discussion

### 3.1 Resin Synthesis and Characterization

VA and cardanol (C) were chosen to be the phenolic constituents in the synthesis of the liquid resin for a biobased epoxy resin. Phenolic coupling took place via an electrophilic aromatic condensation reaction without the use of formaldehyde. Formaldehyde is typically used in resole/novolac type phenolic coupling reactions, but the presence of the hydroxymethyl group within VA provides the reactivity necessary in the presence of a strong acid catalyst without any aldehyde.^45^ The reaction progress was tracked using APC to monitor the consumption of the VA and simultaneously allow a certain extent of oligomerization of the material until all of the VA was consumed. The APC chromatograms of the VAC as a function of reaction time are presented in Figure 1. Full consumption of VA through the coupling reaction took around 26 h at 90 °C. 10% molar excess of C was kept in the mixture as a reactive diluent to reduce viscosity and to obtain anticipated flexibility for surface coatings. Figure 1 also shows that three major products, besides free C, formed during the coupling reaction, which is attributed to the ortho- and para-substitution capabilities of the phenolic moiety of C. The product was found to have 45 wt % mono-substituted cardanol, but there was also a significant amount of di- (24%) and tri- (11%) substituted C, making this liquid resin a mixture of oligomeric and monomeric species. The representative chemical structures of the higher molecular weight oligomers are listed in Figure 1. The reaction was kept until all the free VA was consumed, yielding 10% by weight of excess free cardanol that is kept in the mixture as a diluent. Further reaction times after the VA is fully consumed cause gelation of the product; thereby, the reaction should be monitored carefully. The ^1^H- and ^13^C NMR of VAC presented in Supporting Information Figure 1 show the successful coupling of VA with C via the formation of the alkyd −CH_2_ bonds between 3.7 and 3.9 ppm on the ^1^H NMR spectrum (Supporting Information Figure 1­(a)) and 66.0 ppm on the ^13^C NMR spectrum (Supporting Information Figure 1­(b)).

**Figure 1:**
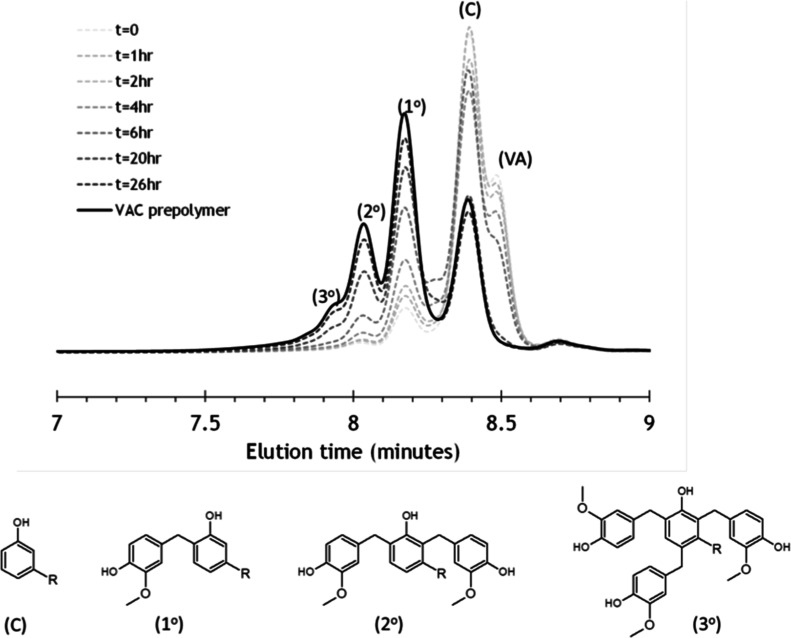
Reaction progress of VA-cardanol (C) coupling to synthesize the liquid resin VAC (R/C_15_H_31–*n*_, *n* = 0, 2, 4, and 6).

The epoxidation of VAC was performed in the presence of excess epichlorohydrin and the TEAB catalyst. Supporting Information Figure 2 compares the APC chromatograms of EVAC with the precursor VAC. The increase in molecular weight due to the addition of oxirane units is confirmed via the shifting of all four APC peaks by roughly 0.02 min. The ^1^H- and ^13^C NMR spectra of the EVAC are also detailed in the Supporting Information (Supporting Information Figure 3a,b), confirming the addition of oxirane units to the VAC via the formation of the new peaks between 2.5 and 4.5 ppm on the ^1^H spectrum and 40–70 ppm on the ^13^C NMR spectrum, respectively. The EEW of the EVAC was measured as 300 ± 10 g/equiv via titrations, which is close to the theoretical value of 280g/eq (factoring the extent of oligomerization), confirming the great extent of oligomerization and formation of a negligible amount of further oligomeric species during the epoxidation process. Based on the APC and NMR data, the degree of oligomerization was measured to be 0.35 for EVAC. The ^1^H- and ^13^C NMR spectra of the DFDA curing agent are also presented in Supporting Information Figure 4­(a,b), respectively. The purity of DFDA was determined as 98% via the integration ratio of peak number 1 (1H) to number 3 (2H) of the ^1^H NMR spectrum upon adjusting the number of protons representing each peak.

Phenolic epoxy resins, such as BPA derivatives, possess relatively high viscosities and poor processability. Thus, they are generally diluted with low-viscosity monofunctional epoxy monomers such as C12–C14 alcohol glycidyl ethers.^46,47^ On the other hand, the synthesized EVAC has various side chains and branches inherited from C and VA, along with 10 wt % free C glycidyl ether upon epoxidation, all assisting in producing a liquid epoxy resin with a relatively low viscosity at 25 °C. The viscosities of the EVAC-DGEBA blends at 25 °C are depicted in Table 1, showing the reduction in the viscosity of DGEBA from 11,000 cPs to 314 cPs with the addition of EVAC. Viscosity of this resin is significantly lower than that of its commercially available phenolated counterparts, NC-514 diepoxy resin and epoxidized cardanol-formaldehyde resin (NC-547), despite having a similar extent of oligomerization.^48^

**Table 1:** Summary of Liquid Resin Blend Viscosities and Cured Resin Extents of Cure Values^a^

epoxy blend (EVAC/EPON 828) (wt %)	blend viscosity at 25 °C, (cP)	gel time with DFDA (@25 °C, hours)	extent of cure, (%) (DFDA)	extent of cure, (%) (Epikure W)
100% EVAC	314	7.65	99.9/98.2	99.8/98.8
67/33	617	7.01	99.7/99.3	99.9/99.6
50/50	1040	5.98	99.1/99.4	99.7/98.9
33/67	1800	4.24	98.9/99.1	99.8/99.0
100% EPON 828	11000	3.77	98.6/99.6	99.9/99.2

a First value in the extent of cure data is based on epoxy conversion, with the second value based on amine conversion.

### 3.2 Extent of Cure and Gel Time

The extent of cross-linking between the epoxy blends and the curing agents was determined using NIR spectroscopy based on the changes in spectral data resulting from the conversion of epoxy and amine groups through the thermal cure processing. The overlay of the NIR spectral data of the pre- and postcured epoxy blends with DFDA and Epikure W is shown in Supporting Information Figures 5­(a,b), respectively. The oxirane rings of EVAC absorb at about 4530 cm^–1^ and 6060 cm^–1^. The absorbances at 4925 cm^–1^ for DFDA and 5025 cm^–1^ for Epikure W are characteristic of primary amine absorption, while both primary and secondary amines absorb at approximately 6600 cm^–1^. All resins were determined to have extents of cure >98% with respect to epoxy conversion and >99% with respect to amine conversion, which indicates the almost full conversion of the epoxy and amine groups after a given cure cycle, as shown in Table 1.

The gel time at 25 °C was measured only for the epoxy blends cured with DFDA, and the results are included in Table 1. Since Epikure W is an aromatic amine, it reacts slowly with the epoxy resin at ambient temperatures and usually requires elevated temperatures to assist in timely polymerization, while DFDA is an aliphatic amine and cures at ambient conditions in shorter time periods. The gel time results for epoxy blends with DFDA show that the addition of the EVAC to DGEBA slowed down the curing process. For example, the neat DGEBA-DFDA mixture gelled within 220 min while neat EVAC-DFDA took almost 450 min. This is expected since EVAC is a larger molecule with long side chains and branches, creating steric limitations and various other electronic effects, which slow down the gelation process.

### 3.3 Thermomechanical Characterizations

#### 3.3.1 Thermogravimetric Analysis

The TGA thermograms for the cured epoxy blends with DFDA and Epikure W are shown in Figure 2a,b, respectively, where the weight of each sample changes as a function of temperature. The thermal properties are recorded in Table 2 corresponding to the temperatures at 10% (*T*_10%_) and 50% (*T*_50%_) weight loss, the peak temperature of the weight loss rate (*T*_max_), and char content (wt %) at 700 °C under a N_2_ atmosphere. All fully cured blends showed three major degradation steps, regardless of the test environment and the curing agent. The first step of degradation observed between 295 and 350 °C is attributed to the degradation of aliphatic segments and the side chains, since they represent dangling chain ends in the formed polymer networks. The second stage degradation observed between 350 and 430 °C is assigned as the degradation of more rigid epoxy-amine bonds and cross-links, while the final degradation occurring above 450 °C is attributed to the degradation of the aromatic segments.

**Figure 2:**
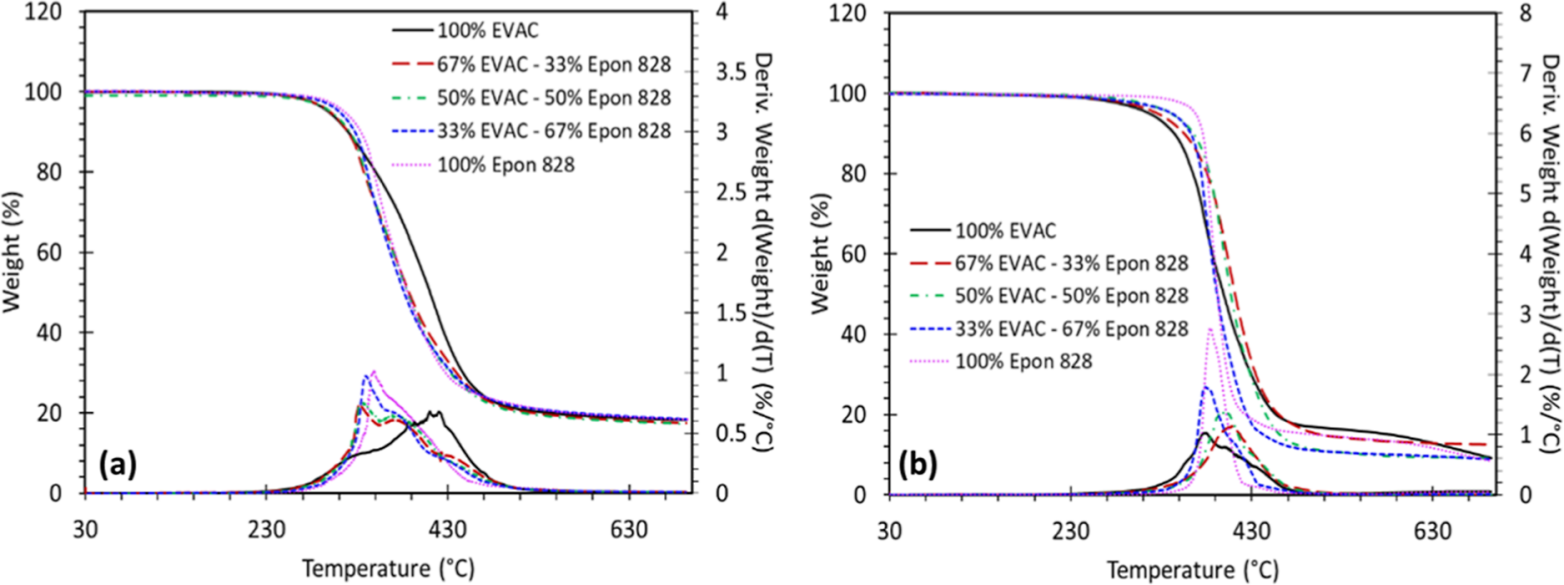
TGA results for blends cured with (a) DFDA and (b) Epikure W tested in an inert (N_2_) environment.

**Table 2:** Summary of the Thermal Stability in an Inert (N_2_) Atmosphere as Obtained via TGA

epoxy blend (EVAC/EPON 828) (wt %)		*T*_10%_ (°C)	*T*_50%_ (°C)	*T*_max_ (°C)	char (@700 °C) (wt %)
curing agent DFDA	100% EVAC	297 ± 1.2	413 ± 4.6	416 ± 3.6	18.8 ± 1.5
	67/33	300 ± 0.55	387 ± 0.58	372 ± 2.5	17.5 ± 0.12
	50/50	302 ± 2.6	384 ± 2.1	368 ± 7.5	17.5 ± 1.1
	33/67	308 ± 2.3	382 ± 1.3	339 ± 1.7	18.5 ± 0.61
	100% EPON 828	316 ± 0.9	386 ± 0.81	348 ± 1.7	18.4 ± 0.93
curing agent Epikure W	100% EVAC	321 ± 3.8	396 ± 2.9	382 ± 2.5	6.57 ± 0.7
	67/33	325 ± 1.4	413 ± 2.9	406 ± 2.9	8.09 ± 0.27
	50/50	339 ± 4.7	407 ± 0.76	402 ± 1.0	9.93 ± 0.67
	33/67	358 ± 2.1	394 ± 0.03	380 ± 1.0	8.08 ± 0.63
	100% EPON 828	368 ± 0.17	392 ± 0.34	382 ± 0.19	8.3 ± 0.32

With both curing agents, increasing the EVAC content slightly decreased *T*_5%_, which can also be considered the temperature at which the sample begins to degrade. This reduction is attributed to the cardanol C15 side chain since long aliphatic groups represent the dangling chain ends that tend to degrade first upon heating. Conversely, for epoxy blends cured with DFDA, 100% EVAC content gives the highest *T*_50%_ and *T*_max_, indicating a lower degradation rate. For blends cured with Epikure W, samples with 67% and 50% EVAC have the highest *T*_50%_ and *T*_max_, while 100% EVAC and 100% EPON-828 exhibit the highest degradation rates. Despite a decrease in initial decomposition temperature, the combination of 100% EVAC cured with DFDA provides the most improved degradation rate when compared to the use of Epikure W as the curing agent. Additionally, a significant improvement in the char content was observed for all cured epoxy blends with DFDA versus Epikure W. The improved degradation rates and char yield are due to the furanic nature of DFDA. DFDA starts to degrade sooner than aromatic Epicure W due to the lack of highly electron-dense and rigid phenyl moieties and the presence of an extra aliphatic methylene linkage between the amine and furan groups. However, the furan moieties transform into a more stable form during degradation; ultimately, improving the rate of degradation and the char yield of the network at elevated temperatures.^36^ In addition, highly electron-dense double bonds located on the aliphatic side chain of EVAC can be another reason for the improved rate of degradation of EVAC-containing cured blends that were cured with DFDA. In addition, EPON-828 is derived from BPA, which possesses a propylene bridge between two phenyl moieties, while EVAC is similar to BPF, possessing a methylene bridge between the phenyl moieties. The CH_3_ moieties on EPON-828 may act as chain ends in the thermoset network and start to degrade sooner upon heating relative to EVAC.^49^

#### 3.3.2 Dynamic Mechanical Analysis

DMA was used to investigate the thermophysical properties of the cured epoxy resins. This includes cross-link density (ν), molecular weight between cross-links (*M*_c_), glassy modulus (*E*′ @ 25 °C), and glass transition temperature (*T*_g_), as obtained from the maxima of loss moduli and tan δ thermograms. Temperature dependence of the *E*′ and *E*″ for the cured epoxy resins is presented in Figure 3. The temperature dependences of tan δ for the cured epoxy resins are also presented in Supporting Information Figure 6. The cross-link density values of the cured epoxy blends presented in Table 3 show a reducing trend with the EVAC addition for both Epikure W and DFDA curing agents. This is due to the bulky C15 alkyd chain of the EVAC molecule, which assists in imparting more free volume within the formed polymer network. In addition, blends cured with Epikure W yielded higher cross-link density values relative to those cured with DFDA. This is attributed to the methylene units between the furan rings and amine groups on the DFDA molecule, acting as spacers, thereby increasing free volume. In addition, amine groups on Epikure W are located on a single aromatic ring. While the amine groups of DFDA are separated by three methylene and two furan moieties, yielding thermoset networks having higher molecular weight between cross-linked junctions. The density (ρ) values of the cured epoxy blends are included in Table 3 and show an opposite trend to cross-link density, yielding higher values for cured resins containing DFDA. This could be explained via better π–π stacking of furan rings in a gelled thermoset network relative to cyclic and aromatic moieties.^36,50^ Furthermore, as seen in Table 3, the *E*′ of the cured blends is reduced by the usage of EVAC due to the relatively soft segments being incorporated into the polymer network via the side chain of cardanol. In addition, DFDA-containing cured blends show improved glassy moduli compared to those cured with Epikure W, probably due to better π–π stacking. Additionally, restricted rotational motion of the furan rings relative to aromatic ones in a gelled thermoset network could be another factor for the improved glassy moduli of the DFDA-containing cured resins. It is noted that the 100% EVAC-

**Figure 3:**
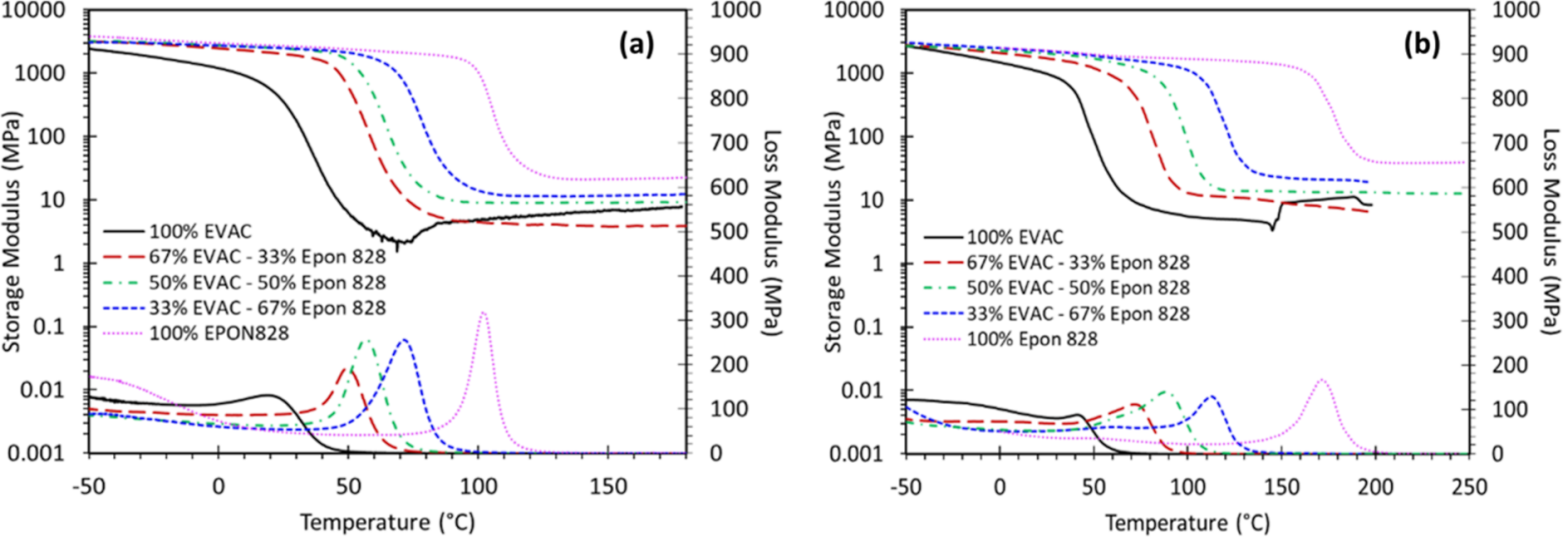
Representative DMA thermograms of the fully cured epoxy-amine blends cured with (a) DFDA and (b) Epikure W.

**Table 3:** Thermomechanical Properties of the Cured Resins

epoxy blend (EVAC/EPON 828) (wt %)		ν (× 106), (mol/m3)	ρ, (g/cm3)	Mc, (g/mol)	*E*′ (at 25 °C), (GPa)	*T*_g_ (E″), (oC)	*T*_g_ (tan δ), (oC)	*T*_g_ (DSC), (oC)
curing agent DFDA	100% EVAC	216	1.10 ± 0.008	5114	0.36 ± 0.03	19 ± 1.1	41 ± 0.5	41 ± 1.4
	67/33	348	1.15 ± 0.002	3304	1.76 ± 0.28	49 ± 1.6	62 ± 1.0	50 ± 1.4
	50/50	628	1.17 ± 0.007	1856	2.48 ± 0.05	56 ± 0.5	67 ± 0.4	55 ± 0.6
	33/67	1011	1.18 ± 0.006	1165	2.61 ± 0.14	71 ± 0.4	81 ± 0.4	68 ± 0.4
	100% EPON 828	2071	1.20 ± 0.006	580	2.73 ± 0.08	102 ± 0.6	107 ± 0.5	108 ± 0.9
curing agent Epikure W	100% EVAC	935	1.09 ± 0.008	1165	0.93 ± 0.10	40 ± 2.5	55 ± 2.6	49 ± 1.2
	67/33	1511	1.12 ± 0.005	742	1.75 ± 0.04	71 ± 0.7	84 ± 0.6	80 ± 0.4
	50/50	1589	1.13 ± 0.007	711	2.16 ± 0.11	87 ± 2.7	99 ± 3.0	91 ± 0.1
	33/67	2389	1.14 ± 0.003	480	2.18 ± 0.04	113 ± 0.5	122 ± 0.5	124 ± 0.4
	100% EPON 828	3315	1.16 ± 0.002	351	2.20 ± 0.18	172 ± 1.9	182 ± 1.0	186 ± 1.1

containing fully cured resins exhibited an abrupt rise in their rubbery moduli values immediately upon relaxing out of the glass transition upon heating. This is likely a measurement artifact resulting from sample deformation during testing.

The *T*_g_ values of the cured epoxy blends are listed in Table 3. Increasing EVAC content resulted in a reduction in the *T*_g_ due to the formation of cured resins possessing reduced cross-link densities, regardless of which curing agent was utilized. The decrease in these properties is caused by the plasticizing effect of the C15 alkyd chain from the cardanol moiety on the EVAC structure.^45,51^ A lower *T*_g_ is a good indication of improved flexibility and room temperature (RT) cure as a thin film. On the other hand, Table 3 also shows that Epicure W-containing networks demonstrated higher *T*_g_ values than those containing DFDA, while the cured resins containing DFDA yielded higher *E*′ values compared to those containing Epikure W, irrespective of the epoxy resin utilized. The *T*_g_ values obtained from the tan δ maxima show similar trends to the *T*_g_ values obtained from the *E*″, yielding higher numbers for Epikure W and a clear reduction in *T*_g_ as EVAC is incorporated into the system (see Supporting Information Figure 6­(a,b)). The *T*_g_ of the phenolated epoxidized cardanol resins (NC-514) was found to be around 50 °C, slightly above EVAC upon thermal curing with an aromatic polyamine isophane diamine. The difference in *T*_g_ is proposed to be due to the side chain cross-linking of NC-514 upon thermal cure since the direct phenolation of cardanol happens from the side chain unsaturation sites. In addition, thermally cured samples of EPON-828 with DFDA demonstrated impressive char yields around 25% (@ 800 °C in N_2_) and high storage modulus (*E*′) with EPON 828 close to 3.0 GPa, much higher than cycloaliphatic counterpart PACM (i.e., 2.2 GPa and <5% char).^36,52^

#### 3.3.3 Differential Scanning Calorimetry

The *T*_g_s of the cured resins were also measured using DSC. Supporting Information Figure 7­(a,b) shows the DSC thermograms of the thermosets cured with DFDA and Epikure W, respectively. Results are also listed in Table 3. For these cured resins, *T*_g_ decreases as the EVAC content increases, agreeing with the DMA results.

#### 3.3.4 Tensile Tests

Dog-bone-shaped tensile bars were tested to obtain the (a) Young’s Modulus (*E*), (b) tensile strength (σ), and (c) tensile strain of the cured resins. Results are listed in Figure 4. The addition of flexible EVAC did not result in a significant reduction in *E* and σ up to 50 wt % content for DFDA, while these values slightly increase for the resins cured with Epikure W, again up to 50 wt % EVAC content; although with no statistical difference. Above 50 wt % EVAC content, expected reductions in *E* and σ are clearly observed for all cured resins. It is believed that the initial *E* and σ values of these cured resins are due to the rigidity of the EPON-828. The addition of EVAC leads to a plasticization effect within the cured thermoset network. In addition, up to 67 wt % EVAC, DFDA-containing cured resins yielded slightly higher moduli than those containing Epikure W; however, with little to no statistically different significance. These slightly higher values may be due to the furanic nature of the DFDA, which may promote better π–π stacking relative to the Epikure W. However, neat EVAC cured with DFDA demonstrated reduced *E* and σ values upon curing relative to the neat EVAC cured with Epikure W. As seen in Figure 4(c), tensile strains of the formulations increase slightly with an increase in EVAC content. Interestingly, the 100 wt % EVAC-DFDA cured resin possesses a strain value of about 20%, potentially resulting from the fact that, at 25 °C, the cured resin is well within its glass transition.

**Figure 4:**
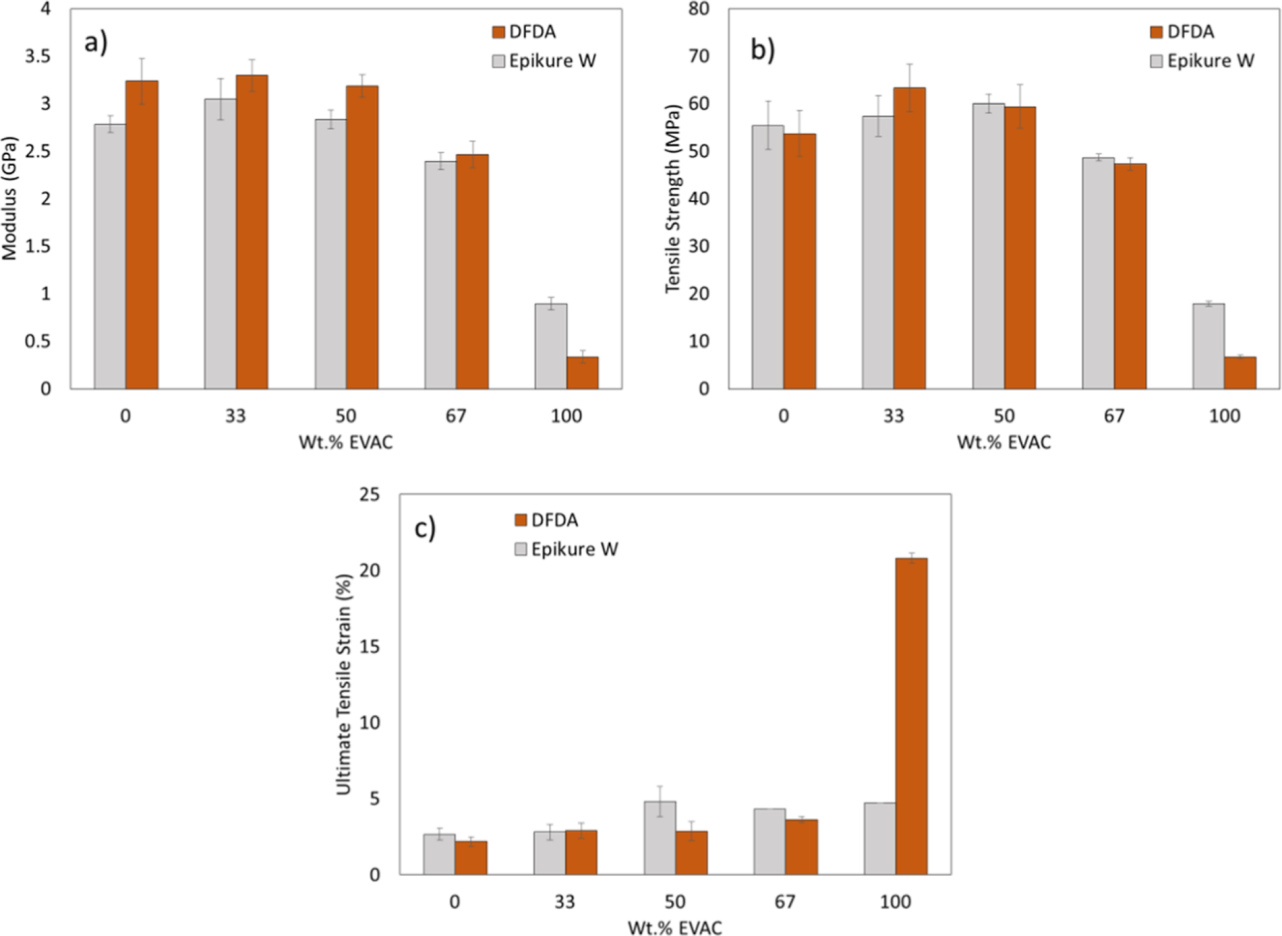
Summary of the mechanical test results: (a) Young’s Modulus (*E*), (b) tensile strength (σ), and (c) tensile strain as a function of EVAC content.

#### 3.3.5 SEM Images

Fractured tensile bars were used to obtain SEM images to gain some insight into the morphology of the cured resins. The SEM images in Supporting Information Figure 8 show smooth and plain morphologies for all of the cured resins, with no evidence of phase separation, indicating high compatibility among the epoxy and amine precursors and the formed thermoset networks.

#### 3.3.6 Contact Angle Measurements

The surface hydrophobicity of the cured resins was studied via sessile water contact angle measurements, with results shown in Figure 5. The shape of the sessile drop on the polymer surface and the contact angle values as a function of EVAC are represented. The incorporation of EVAC into each cured resin formulation increased the water contact angle due to the hydrophobic and nonpolar C15 side chain of the EVAC. At each EVAC wt % formulation, contact angles are not strongly influenced by the curing agent utilized, suggesting the stronger influence of the EVAC component on the surface hydrophobicity of the cured resins. The contact angle values of the EVAC and DFDA (110°) and Epikure W (105°) systems were also found to be slightly higher than the phenol-based tetra-epoxy and aniline-based amine systems reported in the literature (90°).^53^

**Figure 5:**
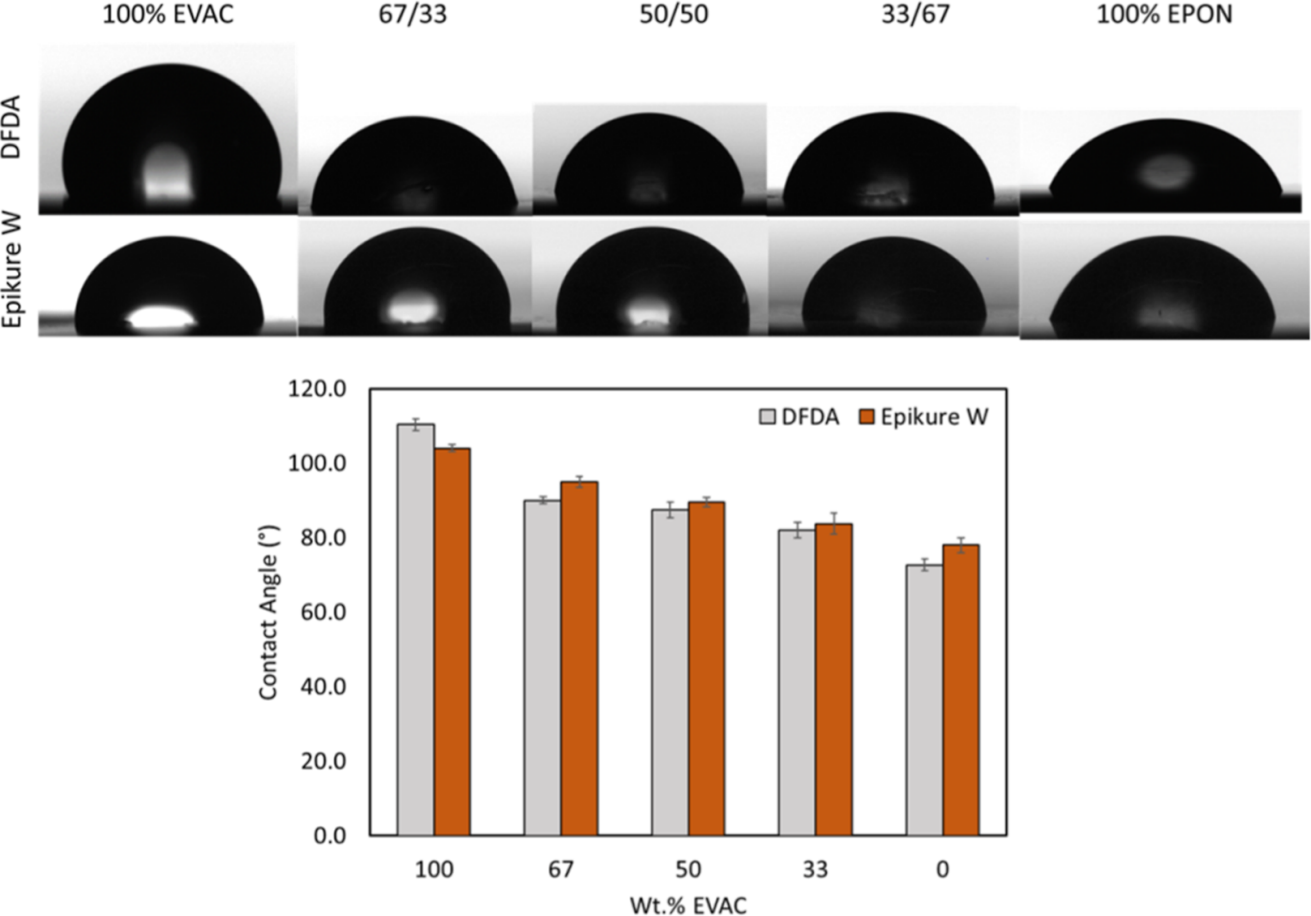
Representative shapes of the sessile water droplet on the surface of DFDA and Epikure W-based post-cured DMA bars (top). Surface contact angle as a function of EVAC content (bottom).

### 3.4 Coating Performance and Properties

Thin films were prepared by applying the precured epoxy-amine liquid mixtures on mild steel panels via a 1 mil drawdown blade. Digital images of the coatings are shown in Figure 6 after various curing schedules. The coatings cured with DFDA at RT for 1 week and cured at 80 °C for 4 h had a glossy appearance and were orange/dark yellow in color. The steel substrate remained visible through these coatings. The coatings cured with Epikure W at RT for 2 weeks and cured at 80 °C for 4 h were also glossy in appearance and light yellow/clear in color. All FC samples were also glossy in appearance, but black and opaque on the substrate. Additionally, all panels that were cured with Epikure W were not set-to-touch after following the intended cure schedule due to the slow cure nature of Epikure W. These panels were left for curing at ambient conditions for an additional week until they were fully nontacky. Furthermore, the neat EVAC-Epikure W system required an additional 2 weeks until set-to-touch at ambient was achieved.

**Figure 6:**
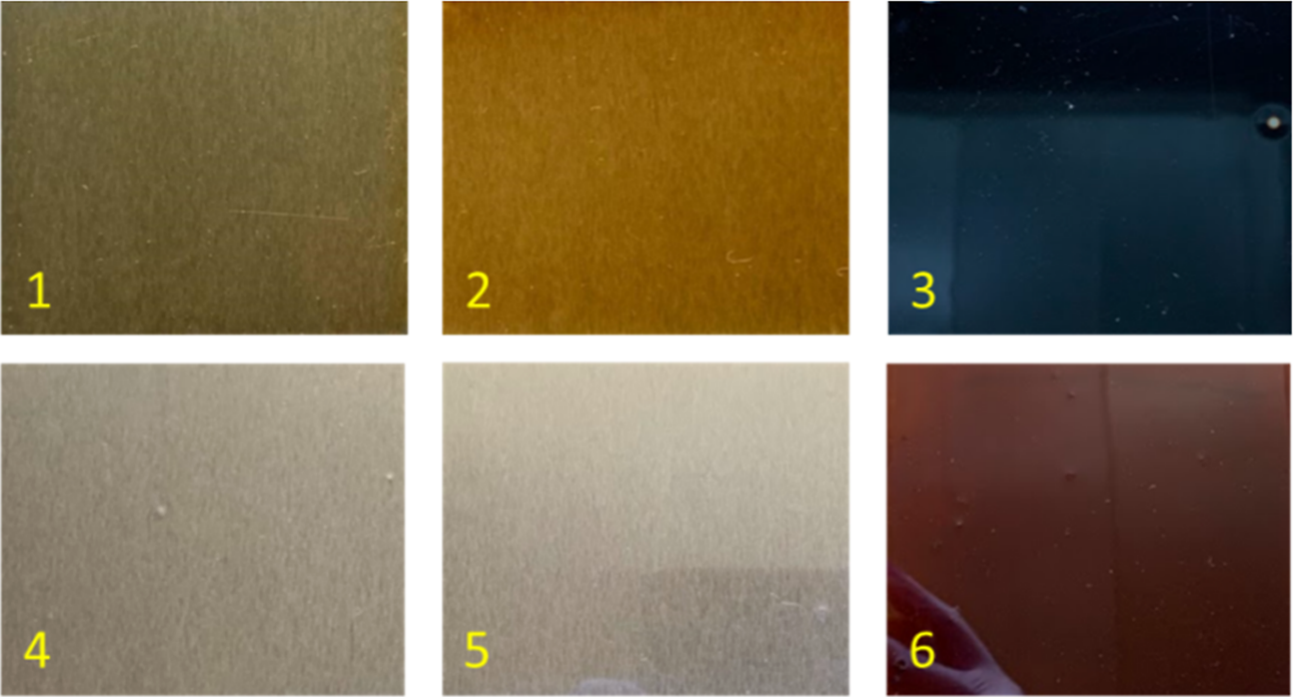
Representative photos of the postcured panels for 50% EVAC-EPON-828 content: (1) DFDA, RT cure, (2) DFDA 80 °C,4 h, (3) DFDA, FC, (4) Epikure W, RT cure, (5) Epikure W, 80 °C, 4 h, and (6) Epikure W, FC.

The gloss measurements are summarized in Table 4 in gloss units (GU) at 20°, 60°, and 85° angles, which are the optimal angles for the high gloss coatings. All the FC coatings demonstrated reduced gloss because of the oxidation at high curing temperatures (i.e., 160–180 °C); however, glosses are still well above 100 GU. Reducing the cure temperature to 80 °C limited the oxidation and increased the gloss for the 80 °C, 4 h cured resins to above 120 GU. Ambient cured resins demonstrated the highest gloss above 140 GU. The high gloss is due to the transparency of the cured resins when ambiently cured, and the metallic surface of the steel substrate remains visible beneath the polymer coating. Metallic materials have a much higher RI and can have a measurement well above 100 GU. In this case, the combination of a high-gloss, transparent polymer coating with a naturally reflective metallic substrate creates a surface with very high gloss. High gloss is an indication of uniform and homogeneous film formation with no phase separation, blushing, blooming, and fisheyes.

**Table 4:** Summary of Gloss Measurements for Cured Obtained at 20°, 60°, and 85° Angles

		gloss (20, 60, 85°)		
epoxy blend (EVAC/EPON 828) (wt %)		ambient (1wk)	80 °C (4 h)	force cure
curing agent DFDA	100% EVAC	(134°, 132°, 99°)	(120°, 112°, 100°)	(114°, 107°, 101°)
	67/33	(134°, 124, 100°)	(132°, 126°, 101°)	(104°, 105°, 100°)
	50/50	(139°, 123°, 100°)	(138°, 124°, 101°)	(115°, 109°, 101°)
	33/67	(137°, 124°, 100°)	(132°, 122°, 100°)	(117°, 108°, 101°)
	100% EPON 828	(135°, 121°, 100°)	(125°, 152°, 100°)	(126°, 112°, 101°)
curing agent Epikure W	100% EVAC	(142°, 164°, 99°)	(133°, 126°, 100°)	(103°, 105°, 100°)
	67/33	(142°, 135, 98°)	(144°, 158°, 101°)	(106°, 108°, 101°)
	50/50	(139°, 127°, 100°)	(141°, 129°, 101°)	(113°, 108°, 101°)
	33/67	(144°, 157°, 100°)	(148°, 152°, 101°)	(121°, 110°, 101°)
	100% EPON 828	(144°, 149°, 94°)	(154°, 166°, 100°)	(109°, 109°, 101°)

Hardness of the coatings was tested via Shore D, and the results are shown in Figure 7. The curing schedule had a slight effect on Shore D hardness. However, overall, as EVAC content increased, the hardness decreased, with such a performance trend attributed to the flexibility of the aliphatic side chain of EVAC.

**Figure 7:**
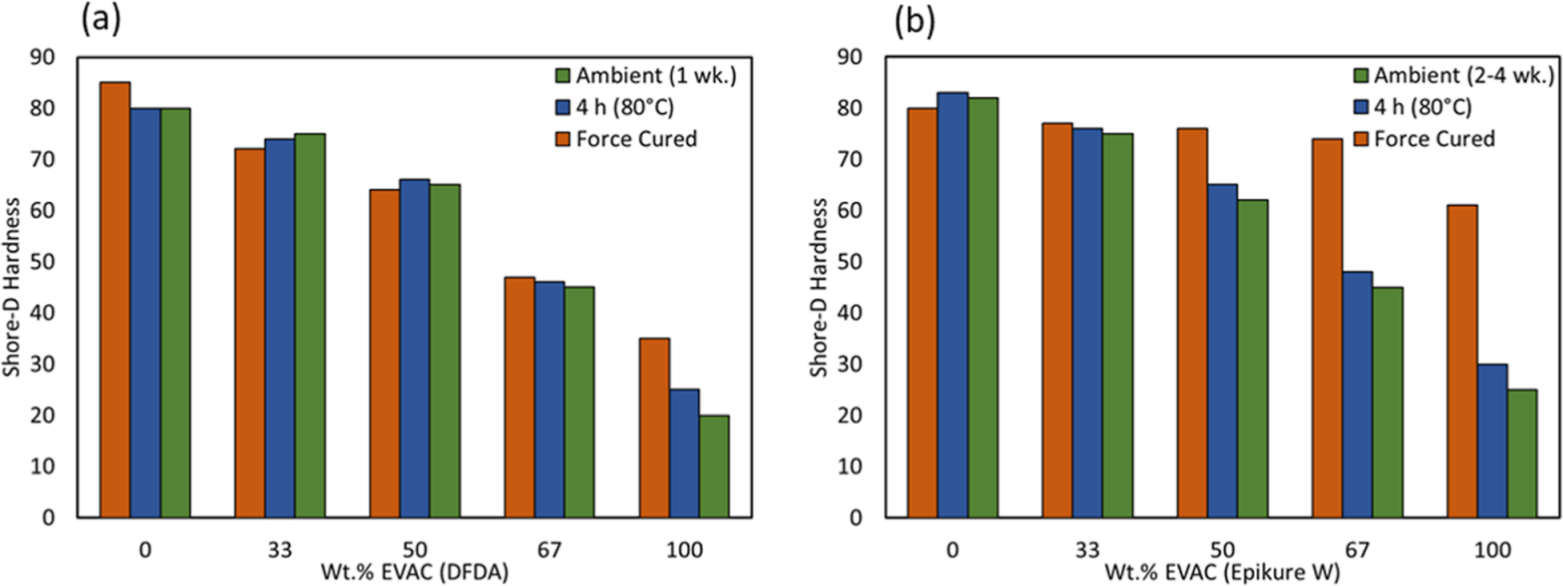
Shore D hardness as a function of EVAC content for various cure schedules: (a) DFDA and (b) Epikure W.

Adhesive properties of the coatings were evaluated via the cross-hatch adhesion and pull-off test, and results are summarized in Table 5. The adhesion mechanism of epoxies is a complex phenomenon, and the hydroxyl groups play a large role in adhesive strength to metallic substrates.^54^ The curing reaction of epoxies results in the formation of hydroxyl groups from the ring opening reaction of the epoxide. The ether and hydroxyl groups form hydrogen bonds with the metal oxide groups of the substrate and serve as the main force of adhesion.^55^ For the epoxy systems cured with both DFDA and Epikure W, the force-cured samples exhibited the lowest adhesion to the steel substrate. This could be due to an increase in internal stress from the differences in the thermal expansion coefficients between the cured resin and the steel panel.^56^ Additionally, the force-cured samples were heated well above their respective *T*_g_s and then cooled slowly back to ambient temperature. Above the *T*_g_, molecular reorientation can occur - relaxing the internal stress, but below *T*_g_, these reorientations cannot occur, and internal stresses are developed.^57^ Coatings cured at ambient conditions and at 80 °C for 4 h exhibited improved adhesive strength. Additionally, coating formulations cured with both amines displayed good adhesion to the metal (5B) for EVAC-rich formulations, and adhesion is completely lost as EVAC is completely replaced with EPON-828. Networks without EVAC (EPON-828 DFDA/Epikure W) are rigid and densely cross-linked; therefore, the presence of EVAC provides the anticipated flexibility derived from the alkyd side chain. Overall, the adhesive properties of the coatings are found to be inversely proportional to the cross-link density and the hardness of the network. In addition, DFDA-containing formulations yielded slightly better adhesion due to their slightly reduced cross-link densities. In addition, the further hydrogen bonding capability of the oxygen atoms within the furans of DFDA could improve the interfacial strength between the coating and the metallic substrate. Phenolic tetra epoxy systems and EPON-828-epoxidized triglyceride cured with polyether amine systems showed similar high adhesion to EVAC-DFDA-based systems between 4B–5B.^53^

**Table 5:** Adhesive Properties of EVAC/EPON- 828 Blends Cured with DFDA and Epikure W Classified via Cross-Hatch and Pull-Off Tests (*Panels Tested after 4 weeks Until Set to Touch)

	curing agent DFDA			curing agent Epikure W		
epoxy blend (EVAC-EPON 828) (wt %)	RT cure	80 °C (4 h)	force cure	RT cure	80 °C (4 h)	force cure
100% EVAC	5B	5B	3B	5B*	5B	1B
67/33	5B	5B	1B	5B	5B	1B
50/50	5B	5B	1B	5B	5B	1B
33/67	5B	5B	1B	1B	0B	1B
100% EPON 828	2B	1B	0B	0B	0B	0B

The cured resin coatings were tested for their impact resistance to evaluate their load distribution properties. The results are summarized in Table 6. Similar to the cross-hatch adhesion test, all force-cured samples performed poorly—most likely due to internal stresses and rigid network formation. Additionally, epoxy blends cured with Epikure W did not perform well unless the system had 100 wt % EVAC content. For samples cured with DFDA (RT 1 week and 80 °C 4 h), impact resistance increased with increasing EVAC content. The reduced impact resistance should be due to the formation of a highly rigid and aromatic network more susceptible to cracking, flaking, and thus delamination of the coating. Alternatively, EVAC provides flexibility from the presence of the long alkyl chain, which absorbs and uniformly distributes the energy under impact; therefore, there is high resistance to impact failure. Overall, the cured resin coatings that were not force-cured and contained a higher percentage of flexible EVAC showed excellent impact resistance similar to adhesion. Impact resistance of the EVAC-DFDA formulations was quite higher than the epoxy-amine systems reported in the literature, which was slightly above 40 lb-ft for DGEBA and polyether amine systems incorporated with epoxidized triglycerides, similar to DGEBA-DFDA systems without any EVAC.^58^

**Table 6:** Impact Resistance (in ft-lb./in) of EVAC/EPON-828 Blends Cured with DFDA and Epikure W Determined from Falling Weight Method (*Panels Tested after 4 weeks Until Set to Touch)

	curing agent DFDA			curing agent Epikure W		
epoxy blend (EVAC-EPON 828) (wt %)	RT cure	80 °C (4 h)	force cure	RT cure	80 °C (4 h)	force cure
100% EVAC	>160	>160	30	84*	>160	12
67/33	60	34	28	16	17	4
50/50	22	24	22	<10	<10	17
33/67	20	24	22	<10	<10	20
100% EPON 828	16	25	15	<10	<10	21

Flexibility of the coatings was determined via a conical mandrel bend test based on qualitative pass or fail classification as summarized in Table 7. All force-cured formulations demonstrated significant cracking and delamination, which correlates to their reduced adhesion and impact resistance. All ambient cured and 80 °C cured DFDA-containing formulations, except 100 wt % EPON-828 content passed the test, while coatings cured with Epikure W only passed the test when a large amount of EVAC was present. In general, flexibility increased with increasing EVAC content, while DFDA-containing formulations yielded improved flexibility compared to the Epikure W-containing formulations.

**Table 7:** Flexibility Performance Determined via Conical Mandrel Bend of EVAC/EPON-828 Blends Cured with DFDA and Epikure W^a^

	curing agent DFDA			curing agent Epikure W		
epoxy blend (EVAC-EPON 828) (wt %)	RT cure	80 °C (4 h)	force cure	RT cure	80 °C (4 h)	force cure
100% EVAC	pass/no cracks	pass/no cracks	pass/minor cracks	pass/no cracks*	pass/no cracks	fail/delamination
67/33	pass/no cracks	pass/no cracks	fail/major cracks	pass/no cracks	pass/no cracks	fail/delamination
50/50	pass/no cracks	pass/no cracks	fail/major cracks	pass/no cracks	pass/minor cracks	fail/delamination
33/67	pass/no cracks	pass/no cracks	fail/major cracks	fail/major cracks	fail/major cracks	fail/delamination
100% EPON 828	fail/major cracks	fail/major cracks	fail/major cracks	fail/major cracks	fail/major cracks	fail/delamination

a Classifications based on visual appearance of coating after test (*Panels tested after 4 weeks until set to touch).

The anticorrosive characteristics of the coatings were evaluated through an elementary environmental test. Coated panels cured at ambient temperature for 1–2 weeks were selected for this study due to their coating performances as described above. Figure 8 contains images of the coated panels after exposure to the noncontrollable environmental conditions for 4 weeks. A significant increase in scribe blistering and corrosion can be observed for EPON-828-rich formulations due to the electrochemical degradation from water and oxygen interacting with the metallic surfaces. Corrosion becomes more predominant as the EPON-828 content increases, most likely due to reduced coating performances in flexibility, adhesion, impact, and surface hydrophobicity. On the other hand, formulations containing DFDA showed improved corrosion performance relative to those containing Epikure W. Like EVAC, DFDA seems to promote flexibility and adhesion of the coatings without increasing the cross-link density and the *T*_g_ of the formed networks. However, DFDA can still promote similar hardness and better modulus values than Epikure W, while yielding a less brittle network. The balance between strength and flexibility should be the key reason for the improved corrosion performance of DFDA-containing coatings.

**Figure 8:**
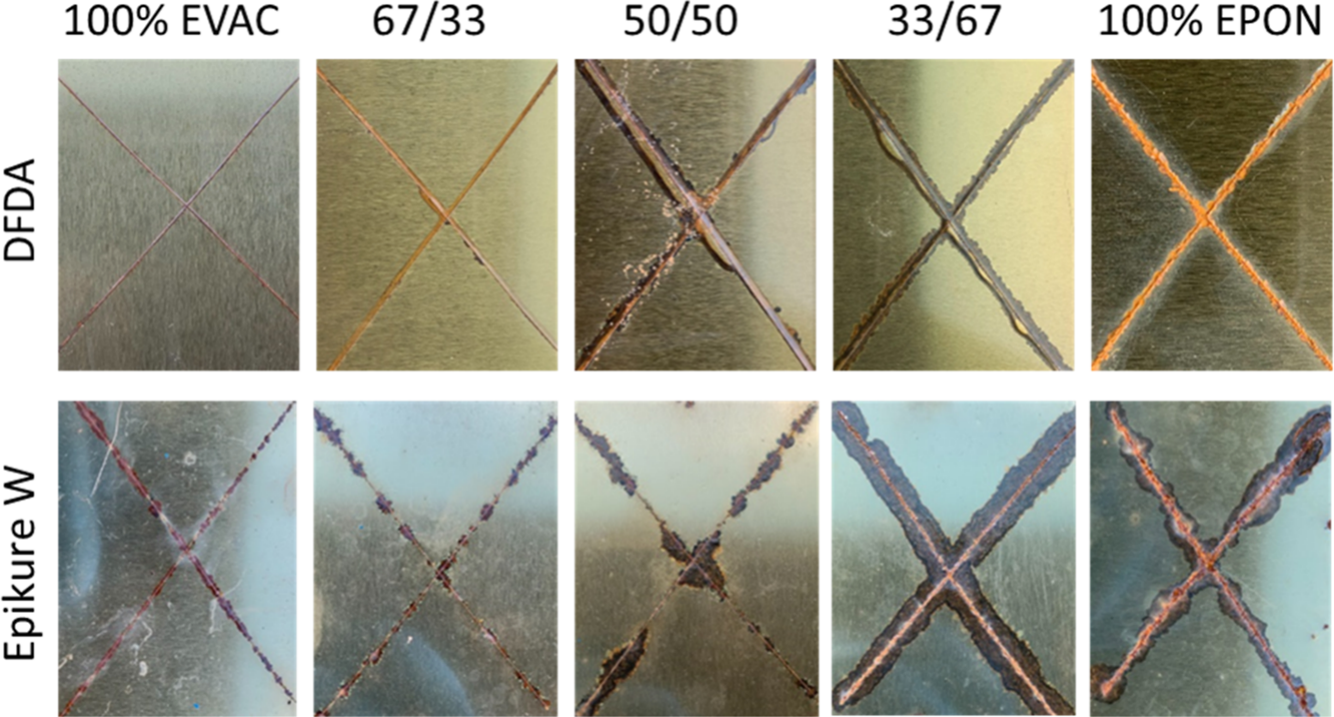
Visuals of coated panels after 4 weeks of exposure to a corrosive environment.

Our environmental resistance results compare well with a similar BPA and aromatic amine system, where 5% nanocoir is loaded, where no water penetration and blisters are observed after salt spray exposure. In addition, the environmental resistance of the cardanol-based benzoxazine system prepared with TETA and co-crosslinked with EPON-828 demonstrated significantly reduced corrosion resistance after exposure to salt spray for 500 h, showing creep and blisters.^32^ ^59^

The anticorrosion performance of EVAC-DFDA coatings is primarily attributed to the formation of a densely cross-linked polymer network that acts as an effective physical barrier. This network significantly limits the ingress of corrosive agents, such as moisture, oxygen, and ions, thereby preventing them from reaching the underlying substrate. The bisphenol-F-based rigid molecular structure of EVAC imparts high chemical resistance to the coating. Alongside the high cross-link density provided by DFDA, this contributes to enhanced durability and environmental stability. In addition, the presence of a C15 alkyl side chain derived from cardanol improves the flexibility, hydrophobicity, and wetting properties of the network, particularly in comparison to conventional BPA-based epoxies. Also, both EVAC and DFDA exhibit superior adhesion properties compared with traditional systems like EPON-828 and EPIKURE-W. This improved adhesion strengthens the interface between the coating and the substrate, which is a critical factor in resisting delamination and corrosion under mechanical or environmental stress. The furan ring within the DFDA structure may behave similarly to an epoxy moiety, facilitating additional hydrogen bonding. This contributes to stronger coating-substrate compatibility and enhances both mechanical resistance and flexibility, further enhancing the coating’s anticorrosive capabilities. The anticorrosion mechanism of EVAC-DFDA is proposed to be driven by its ability to form a robust, impermeable, and adherent network that resists chemical attack, environmental exposure, and mechanical degradation.

## 4 Conclusions

A hybrid, biobased, regulatory-friendly epoxy resin is synthesized via the combination of VA and cardanol without using any toxic formaldehyde, and cured with a furan-based DFDA and aromatic Epikure-W at varying ratios of BPA-based EPON-828. The physical evaluation of the thermally cured formulations demonstrated a reduction in Young’s modulus from 3.2 to 0.45 GPa and *T*_g_ from 102 to 19 °C for DFDA upon full replacement of EPON 828 with flexible EVAC. SEM showed a good compatibility between EVAC and EPON-828; the addition of the former significantly improved the surface polarity, as demonstrated via an increment in contact angle from 80° to 105° for DFDA. Thin films (5 mils) applied on mild steel panels cured at ambient vs elevated temperatures demonstrated the detrimental effect of high temperature cure on the mechanical performance of the coating formulations, without improving the eventual hardness of the coatings. EVAC-DFDA-based thin films demonstrated excellent flexibility, adhesion values (4B–5B), and impact resistance (160+ ft-lb) along with significantly improved environmental resistance under prolonged exposure relative to their petroleum-based phenolic counterparts.

## Supplementary Material



## Notes

NMR spectral and chromatographic data of VAC, NMR spectral data of EVAC, NMR spectral data of DFDA, IR spectral data of the precured and cured epoxy resins, tan δ thermograms, DSC thermograms, and SEM images of the fractured surfaces of the cured epoxy resins (PDF)
