# Supplementary material for: Evaluation and Formulation of Hybridized Biobased Precursors as Anticorrosive Surface Coatings
Source: Biomacromolecules. 2026 Mar 24;27(4):2583–97. doi: 10.1021/acs.biomac.5c02310 (PMC13080967; doi:10.1021/acs.biomac.5c02310)
Supplement: Supplementary file 1 [file bm5c02310_si_001.pdf]

Supplemental Information:

Evaluation and Formulation of Hybridized Biobased Precursors as Anticorrosive Surface Coatings

Sarah A. Salazar[1,2], Emre Kinaci[1,2], Giuseppe R. Palmese[1,2], Joseph F. Stanzione, III[1,2*]

[1]Department of Chemical Engineering, Rowan University, 201 Mullica Hill Rd, Glassboro, NJ 08028, United States

[2]Advanced Materials & Manufacturing Institute (AMMI), Rowan University, 107 Gilbreth Pkwy, Mullica Hill, NJ 08062, United States

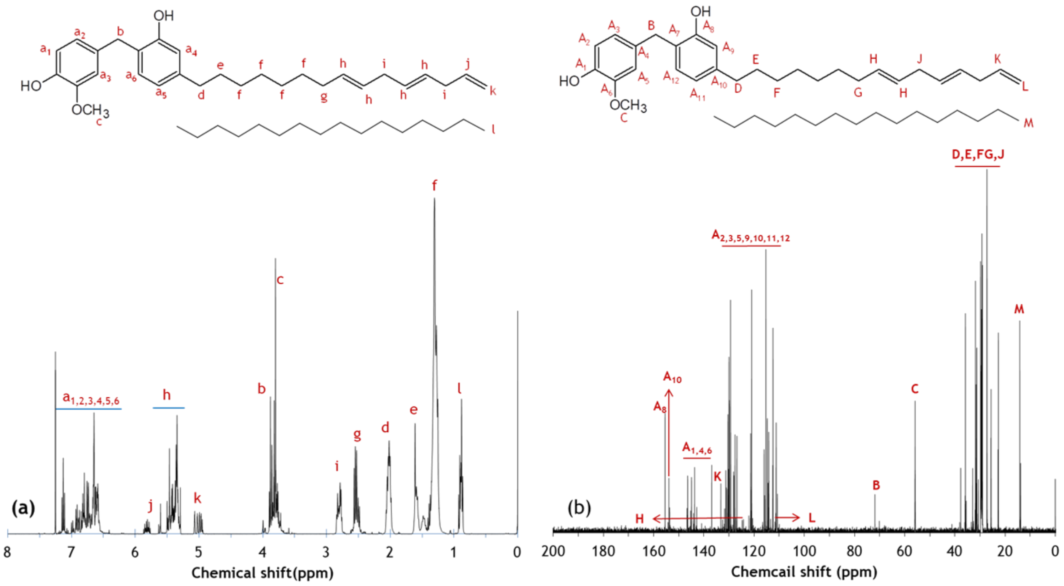

SI Figure 1. Structural verification of VAC via (a) ^{1}H-NMR and (b) ^{13}C-NMR spectra.

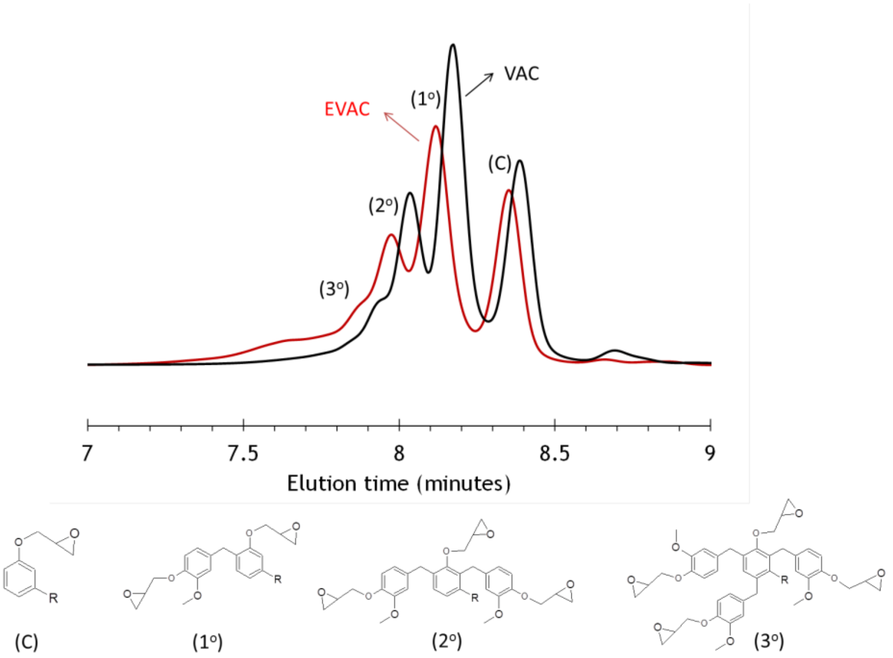

SI Figure 2. APC chromatographic comparison of precursor VAC and product EVAC.

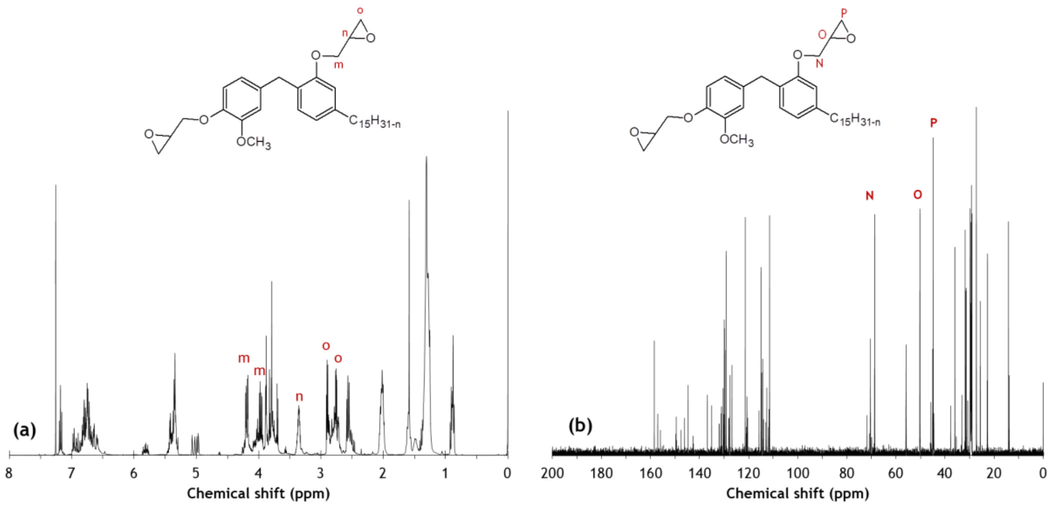

SI Figure 3. Structural verification of EVAC via (a) ^{1}H-NMR and (b) ^{13}C-NMR spectra.

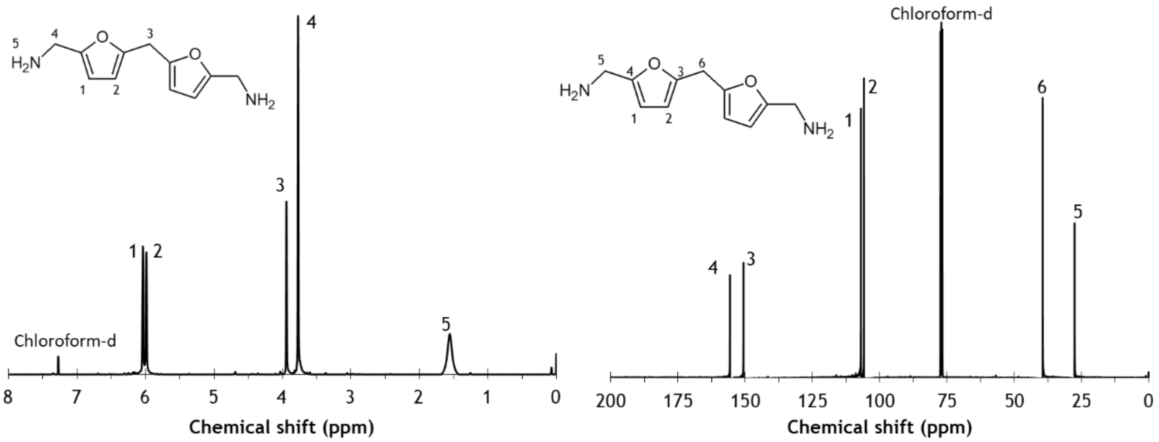

SI Figure 4. ^{1}H-NMR and ^{13}C-NMR spectra of the DFDA curing agent.

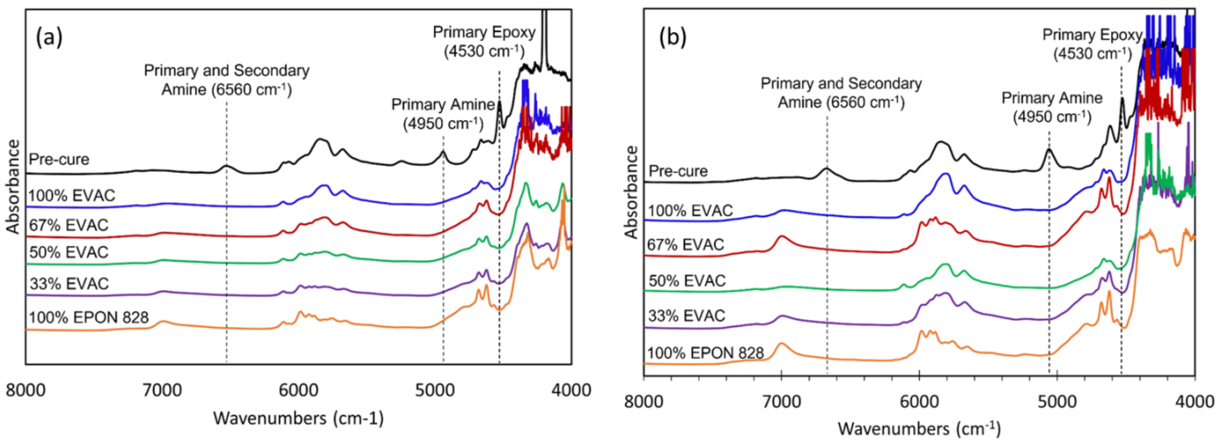

SI Figure 5. Representative mid-IR spectra of Epon-828 – EVAC blends cured with (a) DFDA, and (b) Epikure W.

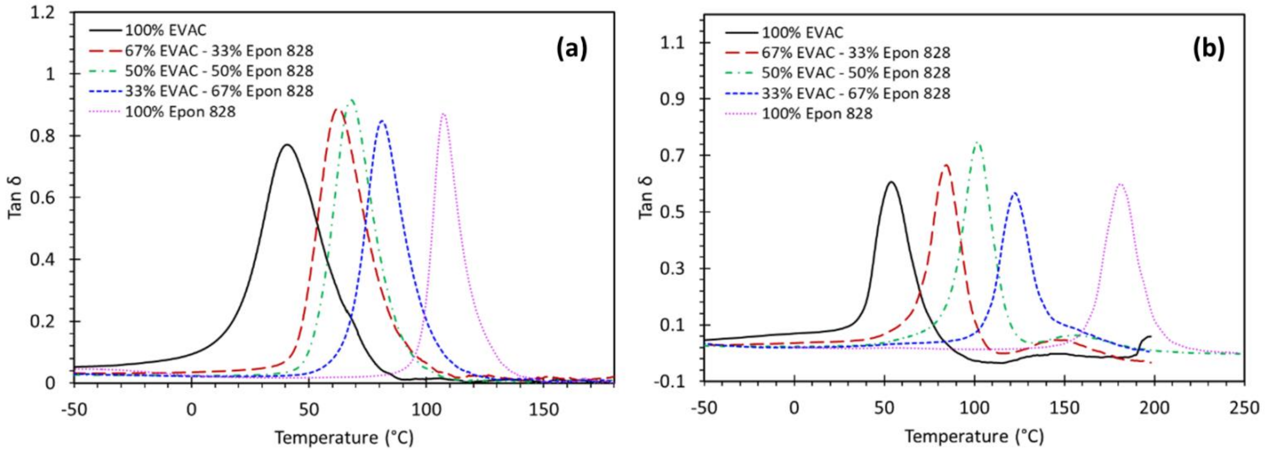

SI Figure 6. Representative tan δ thermograms of the cured resins that were cured with (a) DFDA and (b) Epikure W.

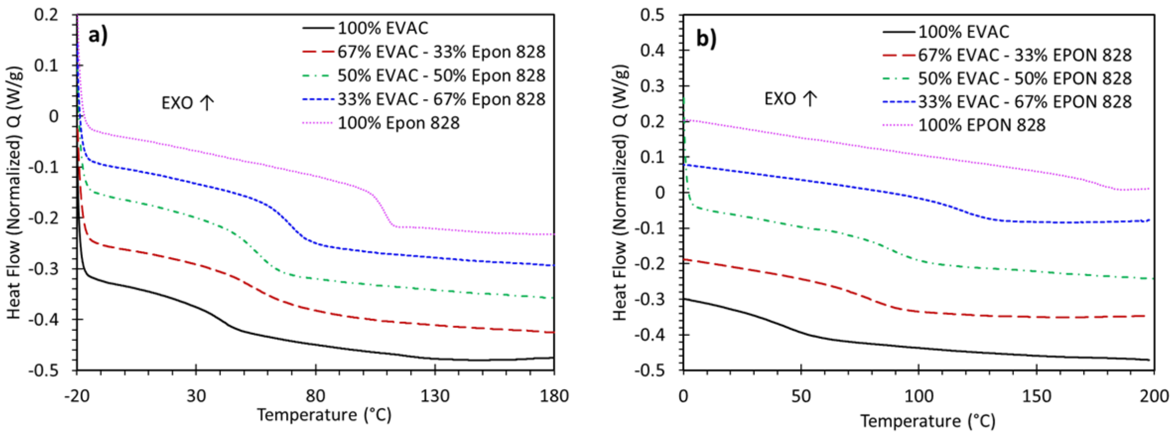

SI Figure 7. Representative DSC thermograms of the Epon 828-EVAC blends cured with (a) DFDA and (b) Epikure W.

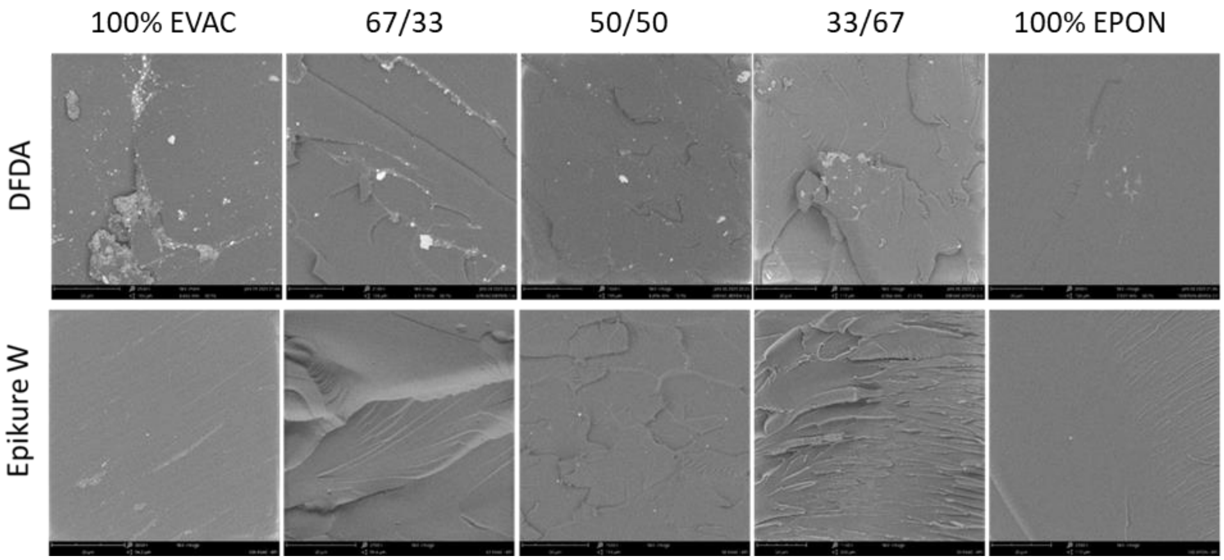

SI Figure 8. SEM images of the cured resins obtained from the fractured surface of tensile bars: curing agent DFDA (top) and Epikure W (bottom).
